# Sea-level rise may not uniformly accelerate cliff erosion rates

**DOI:** 10.1038/s41467-023-44149-3

**Published:** 2023-12-21

**Authors:** M. E. Dickson, H. Matsumoto, W. J. Stephenson, Z. M. Swirad, C. F. Thompson, A. P. Young

**Affiliations:** 1 School of Environment, The University of Auckland, Auckland, New Zealand; 2 Scripps Institution of Oceanography, University of California San Diego, San Diego, USA; 3 School of Geography, University of Otago, Dunedin, New Zealand; 4 Institute of Geophysics, Polish Academy of Sciences, Warsaw, Poland

**Keywords:** Geomorphology, Projection and prediction

**arising from** J. R. Shadrick et al. *Nature Communications* 10.1038/s41467-022-34386-3 (2022)

Shadrick et al.^1^ address the important question of rock coast cliff retreat rates in the face of sea level rise (SLR). Using cosmogenic radionuclide and topographic profile data combined with numerical modelling, they argue to have found a clear causal relationship between the Holocene rate of SLR and the rate of coastal cliff retreat at two coastal sites in Britain. Two of us (Matsumoto and Dickson^2^) developed the rock coast erosion model used by Shadrick et al. and we caution that existing limitations in model process representations prevent accurate projections of hard rock cliff erosion over the next century. We highlight recent process-based studies of cliff erosion that indicate that local coastal response to SLR is likely to be highly variable over decadal time scales.

The geomorphological model used by Shadrick et al.^1^ is an exploratory model to study long-term shore profile evolution. As noted by the authors, it considers a small number of processes using highly abstracted representations^2^. The model time step is equivalent to a single year, which necessitates deep collapsing of the myriad short-term processes that are responsible for cliff and shore platform erosion. This is justified where the intent of modelling is to explore large-scale behaviour and investigate poorly understood phenomena^3^, but the model has limited capability to predict decadal-scale trends. The model appears to have performed well against observed historic retreat rates at Scalby (0.059 and 0.054 m/y)^1^, but a mismatch between observed and modelled rates at Bideford (0.058 and 0.017 m/yr) may relate to limitations in model process representations.

Shadrick et al.^1^ have described a fascinating relationship between Holocene-scale decline in the rate of SLR and a corresponding decline in the rate of cliff erosion. Does it necessarily follow that a decadal-scale acceleration in SLR will result in a corresponding acceleration in cliff erosion rates? The hard-rock sedimentary cliffs studied by Shadrick et al.^1^ have eroded slowly (<~10 cm/yr) over the historical period, but calibrated model forecasts suggest that cliff erosion rates will increase by between 2 to 13 times over the next century. The model used to project future erosion rates was calibrated with observations from a time period with very different SLR rates than those anticipated over the next century. Erosion dynamics at the cliff toe may be quite different under accelerating and decelerating rates of SLR. Understanding this subtlety requires a robust numerical description of wave erosion of rock cliffs, which has proven challenging, because unlike sand beaches, cliff morphology does not respond dynamically to wave forcing. Higher frequency observations of cliff erosion events and the driving processes are required to understand the fundamental processes of cliff erosion, including episodic events and lagged geomorphic response^4^. Recent progress has been made using arrays of wave gauges, repeat laser scanning and cliff-top seismometry^5–8^, but we do not yet have a quantitative process-based description of the relationship between incident wave energy, sea level and cliff erosion rates that is sufficiently detailed to inform robust future predictions of retreat rates. However, it has become clear that abrupt thresholds exist at the cliff toe between wave and water level conditions, implying that future erosion rates are unlikely to ‘simply’ track the future rate of SLR. In some locations, SLR may rapidly transition cliff-toe wave energy spectra from long- to short-wave dominated where a greater proportion of incident wave energy reaches the toe^9^. Significant changes may also occur in the proportion of tidally modulated broken, breaking and unbroken wave impacts^5^. Crucially, it has been shown that breaking wave impacts result in cliff ground shaking (a potential proxy for erosional damage) that is, on average, an order of magnitude higher than for broken and unbroken wave impacts^5^. Hence, it is possible that very fast rates of sea level rise (e.g. RCP8.5 modelled by Shadrick et al.^10^) could (1) transition some cliff toe wave environments to mainly unbroken wave impacts where most wave energy is reflected, and (2) increase the vertical spread of wave-impact energy on the cliff face over time, thereby reducing the amount of time available for cliff destabilisation through notching.

It is plausible, through the processes described above, that accelerating SLR could result in slower cliff erosion rates for some locations. These detailed processes are not included in the morphological model used by Shadrick et al.^1,2^. Further improvements to the model are needed to adequately account for the hydrodynamic transitions that are likely to occur over the next century on rock coasts. In addition, future model improvements should include consideration of sedimentary feedbacks that influence cliff erosion patterns over time and space. For example, historical cliff erosion observations in California show that locations with higher/lower historical cliff erosion rates generally had lower/higher recent erosion rates^11^, and broad-scale numerical modelling in Norfolk, UK has shown that SLR-driven acceleration in cliff erosion rates in one location can lead to beach accretion at another location that decreases future cliff erosion rates^12^. The mass movement of hard-rock cliffs can provide sediment that armours against future cliff erosion. Recent work by Shadrick et al.^10^ have further highlighted the important influence that beaches may have on long-term cliff retreat rates. This effect is not considered in the model, but the presence of gravel and boulder beaches at the base of the study sites described by Shadrick et al.^1^ suggests that the armouring effect cannot be discounted.

Future cliff retreat rates will depend on several factors in addition to the rate of SLR. Changes in wave energy and rainfall are likely to be important^6,7,13^, and geological contingency will exert an overarching control on local-scale cliff and shore platform erosion^14^. In this context, stark contrast in the interpretations of Shadrick et al.^1^ and Swirad et al.^15^ is notable. Both studies are based on cosmogenic nuclide data obtained from Yorkshire sites (Scalby^1^ and Staithes^15^) that have comparable historical erosion rates (5.9 ± 4.3 and 4.5 ± 0.63 cm per year). If each interpretation is acceptable, and if differences in interpretation are attributable to local geological factors^1^, then this highlights the complexity of rock coast evolution and underscores the likelihood that cliff response to SLR will be highly variable, especially at relatively short time scales.

The contribution of Shadrick et al.^1^ is a valuable and welcome addition to the literature and builds significantly upon other work on future SLR and cliff retreat rates^12,16,17^. Their study is the first to project future cliff erosion rates using a morphodynamic model calibrated with cosmogenic radionuclide data. In doing so, the study raises awareness to the prospect that historically stable cliffed coasts may not necessarily be stable in the future. In this correspondence we draw attention to process-based studies that highlight the complex relationship between sea level and cliff erosion. We caution against simplified statements regarding the relationship between SLR and coastal cliff retreat: local coastal response to SLR is likely to vary considerably.

## Data Availability

Data sharing is not applicable to this article as no datasets were generated or analysed during the current study.
